# Safety and efficacy of primaquine in patients with *Plasmodium vivax* malaria from South Asia: a systematic review and individual patient data meta-analysis

**DOI:** 10.1136/bmjgh-2023-012675

**Published:** 2023-12-20

**Authors:** Reena Verma, Robert J Commons, Apoorv Gupta, Manju Rahi, Nitika, Praveen K Bharti, Kamala Thriemer, Megha Rajasekhar, Sauman Singh-Phulgenda, Bipin Adhikari, Mohammad Shafiul Alam, Prakash Ghimire, Wasif A Khan, Rishikesh Kumar, Toby Leslie, Benedikt Ley, Alejandro Llanos-Cuentas, Sasithon Pukrittayakamee, Komal Raj Rijal, Mark Rowland, Kavitha Saravu, Julie A Simpson, Philippe J Guerin, Ric N Price, Amit Sharma

**Affiliations:** 1 ICMR-National Institute of Malaria Research, New Delhi, India; 2 Global Health Division, Menzies School of Health Research, Charles Darwin University, Tiwi, Northern Territory, Australia; 3 WorldWide Antimalarial Resistance Network, Asia Pacific Regional Hub - Australia, Melbourne, Victoria, Australia; 4 General and Subspecialty Medicine, Grampians Health Ballarat, Ballarat, Victoria, Australia; 5 Indian Council of Medical Research, New Delhi, India; 6 Academy of Scientific and Innovative Research (AcSIR), Ghaziabad, Uttar Pradesh, India; 7 Centre for Epidemiology and Biostatistics, Melbourne School of Population and Global Health, The University of Melbourne, Melbourne, Victoria, Australia; 8 WorldWide Antimalarial Resistance Network (WWARN), Oxford, UK; 9 Infectious Diseases Data Observatory (IDDO), Oxford, UK; 10 Centre for Tropical Medicine and Global Health, Nuffield Department of Clinical Medicine, University of Oxford, Oxford, UK; 11 Mahidol Oxford Tropical Medicine Research Unit (MORU), Faculty of Tropical Medicine, Mahidol University, Bangkok, Thailand; 12 International Centre for Diarrhoeal Disease Research, Bangladesh (icddr,b), Dhaka, Bangladesh; 13 Central Department of Microbiology, Tribhuvan University, Kirtipur, Nepal; 14 ICMR-Rajendra Memorial Research Institute of Medical Sciences, Patna, Bihar, India; 15 Department of Infectious and Tropical Diseases, London School of Hygiene and Tropical Medicine, London, UK; 16 HealthNet TPO, Kabul, Afghanistan; 17 Unit of Leishmaniasis and Malaria, Instituto de Medicina Tropical “Alexander von Humboldt”, Universidad Peruana Cayetano Heredia, Lima, Peru; 18 Department of Clinical Tropical Medicine, Faculty of Tropical Medicine, Mahidol University, Bangkok, Thailand; 19 Department of Infectious Diseases, Kasturba Medical College, Manipal Academy of Higher Education, Manipal, Karnataka, India; 20 Manipal Centre for Infectious Diseases, Prasanna School of Public Health, Manipal Academy of Higher Education, Manipal, Karnataka, India; 21 International Centre For Genetic Engineering and Biotechnology, New Delhi, India

**Keywords:** Malaria, Public Health, Systematic review

## Abstract

**Background:**

The optimal dosing of primaquine to prevent relapsing *Plasmodium vivax* malaria in South Asia remains unclear. We investigated the efficacy and safety of different primaquine regimens to prevent *P. vivax* relapse.

**Methods:**

A systematic review identified *P. vivax* efficacy studies from South Asia published between 1 January 2000 and 23 August 2021. In a one-stage meta-analysis of available individual patient data, the cumulative risks of *P. vivax* recurrence at day 42 and 180 were assessed by primaquine total mg/kg dose and duration. The risk of recurrence by day 180 was also determined in a two-stage meta-analysis. Patients with a >25% drop in haemoglobin to <70 g/L, or an absolute drop of >50 g/L between days 1 and 14 were categorised by daily mg/kg primaquine dose.

**Results:**

In 791 patients from 7 studies in the one-stage meta-analysis, the day 180 cumulative risk of recurrence was 61.1% (95% CI 42.2% to 80.4%; 201 patients; 25 recurrences) after treatment without primaquine, 28.8% (95% CI 8.2% to 74.1%; 398 patients; 4 recurrences) following low total (2 to <5 mg/kg) and 0% (96 patients; 0 recurrences) following high total dose primaquine (≥5 mg/kg). In the subsequent two-stage meta-analysis of nine studies (3529 patients), the pooled proportions of *P. vivax* recurrences by day 180 were 12.1% (95% CI 7.7% to 17.2%), 2.3% (95% CI 0.3% to 5.4%) and 0.7% (95% CI 0% to 6.1%), respectively. No patients had a >25% drop in haemoglobin to <70 g/L.

**Conclusions:**

Primaquine treatment led to a marked decrease in *P. vivax* recurrences following low (~3.5 mg/kg) and high (~7 mg/kg) total doses, with no reported severe haemolytic events.

**PROSPERO registration number:**

CRD42022313730.

**WHAT IS ALREADY KNOWN ON THIS TOPIC**

- The WHO South-East Asia Region accounted for ~2% of malaria cases globally in 2022. India accounted for 79% of cases in the region, of which ~40% of cases were caused by *P. vivax*.
- *P. vivax* malaria is a substantial challenge for malaria control and elimination due to relapsing infections.
- Primaquine is the only approved antimalarial to prevent relapses in this region, however, its optimal dosing regimen remains uncertain.

**WHAT THIS STUDY ADDS**

- Individual data from 791 patients enrolled into 7 trials in South Asia were included in a one-stage meta-analysis.
- Aggregated data from 3259 patients from 9 studies in South Asia were included in a two-stage meta-analysis.
- Low and high total dose primaquine regimens appear to have good antirelapse efficacy in South Asia.
- There were no severe haemolytic events with any primaquine dose in patients with ≥30% glucose-6-phosphate dehydrogenase (G6PD) activity.
- The study highlights a relative lack of available data on primaquine efficacy in patients followed for 180 days or more, and a lack of data on tolerability and safety of different primaquine regimens.

**HOW THIS STUDY MIGHT AFFECT RESEARCH, PRACTICE OR POLICY**

- The available data support the current recommendation for low total dose primaquine regimens in South Asia.
- Despite limited data, regimens with higher daily doses appeared to be safe in patients with normal G6PD activity, supporting the introduction of 7 day 0.5 mg/kg primaquine regimens following G6PD testing in the South Asia region.

## Introduction

*Plasmodium vivax* causes an estimated 4.9 million cases of malaria annually with 2.5 billion people at risk of infection.^1^ India is aiming for malaria elimination by 2030, however, in 2020, it was estimated to have contributed 79% of the malaria cases and 83% of malaria deaths to the WHO South-East Asia Region.^1^ The Indian National Center for Vector Borne Disease Control recorded a total of 161 753 confirmed malaria cases in 2021, of which 60 187 cases (37.2%) were caused by *P*. vivax.^2^

Unlike *Plasmodium falciparum*, *P. vivax has* dormant liver stages that can cause relapses of malaria weeks or months later. Relapsing vivax malaria leads to recurrent acute febrile illness, anaemia and morbidity, and contributes to ongoing transmission^3^. Successful treatment of *P. vivax* (radical cure) requires treatment of both the blood and liver stages of the parasite.^3 4^

For more than 70 years, primaquine, an 8-aminoquinoline, has been the only available drug to treat *P. vivax* hypnozoites and prevent vivax relapses. However, primaquine can cause drug-induced haemolysis in individuals with glucose-6-phosphate dehydrogenase (G6PD) deficiency, in addition to other side effects such as gastrointestinal disturbance and methaemoglobinaemia.^4^ Although concerns about haemolysis, coupled with a lack of available bedside G6PD testing, have prevented the widespread implementation of primaquine radical cure,^5^ point of care G6PD testing is increasingly available, with a quantitative test now approved in India, paving the way for safer use of primaquine.^6^

The total mg/kg dose of primaquine is related to antirelapse efficacy, with higher doses of primaquine potentially providing greater efficacy.^4^ The total dose of primaquine used in South Asia is 3.5 mg/kg given over 14 days (0.25 mg/kg/day), in line with the current WHO recommendation.^7^ However, few studies have compared primaquine regimens with higher total doses^8 9^ and a recent individual patient data meta-analysis across all vivax-endemic regions suggested a higher 7 mg/kg total dose may improve efficacy.^10^ Regimens with long durations may have poor adherence, leading to low effectiveness although this is not consistent among all studies.^11 12^ Two recent studies have demonstrated non-inferiority of a 7-day vs 14-day regimen of high total primaquine dose (7 mg/kg).^13 14^ Some countries, such as Brazil, use a lower 3.5 mg/kg total dose regimen over 7 days,^15 16^ which has recently been recommended by WHO.^7^ However, daily dosing may be limited by tolerability and haemolytic safety. Thus, any potential benefits from increasing the total dose or providing an equivalent total dose over a shorter period need to be balanced against the associated risks of haemolysis with higher daily doses.

To better understand the safety, tolerability and efficacy of different total and daily primaquine doses for uncomplicated *P. vivax* in South Asia, we undertook this systematic review and individual patient data meta-analysis of efficacy studies of uncomplicated *P. vivax*.

## Methods

### Search strategy and selection criteria

We systematically searched MEDLINE, EMBASE, Web of Science and Cochrane Central according to Preferred Reporting Items for Systematic Reviews and Meta-Analyses Individual Patient Data (PRISMA-IPD) guidelines (online supplemental checklist S1) to identify prospective efficacy studies of uncomplicated *P. vivax* monoinfection published in any language between 1 January 2000 and 23 August 2021 that were undertaken in South Asian countries including Bangladesh, Bhutan, India, Pakistan, Nepal and Sri Lanka. This work is an extension of a previously published review^17^ with search terms and criteria described in detail in online supplemental box S1. Studies were included if they included a treatment arm with primaquine commencing within 7 days of schizontocidal treatment, had data on age, sex and parasitaemia on day 0, data on schizontocidal and primaquine dosing and reported parasite presence or absence during follow-up.

10.1136/bmjgh-2023-012675.supp1 Supplementary data

Identification of eligible studies was undertaken separately by two investigators (RV and RJC), with disagreement resolved through discussion. Investigators of eligible studies were approached to share data. If available, data from eligible but unpublished studies were also included. Individual patient data were collated in the WorldWide Antimalarial Resistance Network secure repository, where they were standardised into a quality-assured dataset.^18^ Metadata, including study design and study site details, were recorded.

### Procedures

Individual patients’ data were excluded if information on dose and regimen of primaquine were incomplete or if no data were available for baseline parasitaemia, age, sex or weight of the patient. The number of tablets given to each patient were used to calculate primaquine doses. If tablet counts were not available, doses were calculated from age-based or weight-based dosing schemes in the study protocol, or if not available, doses were assumed from planned mg/kg dosing regimens. Total primaquine dose was categorised as very low total dose if <2 mg/kg primaquine, low total dose if 2 to <5 mg/kg corresponding with ~3.5 mg/kg and high total dose if ≥5 mg/kg was given corresponding with ~7 mg/kg. Patients treated with weekly primaquine regimens (0.75 mg/kg/week for 8 weeks) were assessed separately. Primaquine regimens were classified by treatment duration in days into 7-day and 14-day regimens. Daily primaquine mg/kg doses were defined as low daily dose if <0.375 mg/kg/day corresponding with ~0.25 mg/kg/day, intermediate daily dose if ≥0.375 to <0.75 mg/kg/day corresponding with ~0.5 mg/kg/day and high daily dose if ≥0.75 mg/kg/day corresponding with ~1 mg/kg/day were administered.

Parasite transmission was categorised based on subnational data of *P. vivax* incidence per 1000 persons from the Malaria Atlas Project as low (<1), moderate (1 to <10) or high (≥10).^19^ Anaemia was defined as mild (haemoglobin (Hb)≥80 g/L and <110 g/L), moderate (≥50 g/L and<80 g/L) and severe (<50 g/L).^20^ In studies where haemotocrit was measured without Hb, haematocrit was converted to Hb using the formula: haematocrit=5.62+0.26×Hb (g/L).^21^

### Outcomes

The primary efficacy outcomes of this study were the incidence risk of any *P. vivax* recurrence between (1) day 7 and day 42 and (2) between day 7 and day 180. Primary outcomes for haematological safety were a >25% drop in Hb from baseline to an Hb <70 g/L, and an absolute drop in Hb of >50 g/L between day 0 and days 1–14. A composite gastrointestinal endpoint including the presence of vomiting, anorexia, or diarrhoea between days 1 and 14 was the primary tolerability outcome.

The secondary outcomes for haematological safety were the maximum absolute reduction in Hb between baseline and days 1–14, an Hb fall below 50 g/L, renal failure, need for blood transfusion, death (between days 1 and 28) and presence of moderate or severe anaemia as separate outcomes. The secondary tolerability outcomes were the presence of vomiting, nausea, anorexia, abdominal pain, dizziness and diarrhoea as separate endpoints between days 2 and 14, and vomiting within 1 hour of primaquine dosing.

### Statistical analysis

A one-stage meta-analysis of eligible and available individual patient data was undertaken. Cumulative risks of recurrence at day 42 and day 180 were calculated for different primaquine total dose categories by Kaplan-Meier survival analysis. Patients were right censored based on the day of first recurrence, the last visit, a gap of >60 days between blood smears or the last day of study follow-up. Cox regression analyses compared the rate of recurrence between day 7 and day 42 and day 7 and day 180 comparing different primaquine total dose categories to no primaquine after adjusting for sex and age, with shared frailty for study-site. Estimation of incidence rates of recurrent vivax parasitaemia over 180 days were planned for each total dose primaquine regimen, however, no available studies followed individuals through recurrent parasitaemic events.

A two-stage meta-analysis was undertaken in studies with 180 days or more follow-up.^22^ Study-specific aggregated proportions from the one-stage individual patient data meta-analysis were combined with aggregated proportions of patients with vivax recurrence at day 180 from studies that were eligible for inclusion in the one-stage meta-analysis but where data were unavailable. The pooled risk of vivax recurrence at day 180 was estimated using random-effects meta-analysis. Proportions were pooled using the Freeman-Tukey double arcsine transformation, with confidence intervals calculated using exact methods.

Using individual patient data, haematological outcomes were described by primaquine daily mg/kg dose category. A multivariable logistic regression analysis was planned to estimate the effect of primaquine daily mg/kg dose on the odds of the composite gastrointestinal outcome, however, no symptom data were available for the included studies. Comparison of the proportions of acute vomiting within 1 hour were similarly planned between primaquine daily mg/kg dose categories but were not possible.

The effect of heterogeneity in the studies was assessed by removal of one study site at a time from the one-stage meta-analysis, with calculation of the coefficient of variation around day 42 and day 180 cumulative risks. Bias in studies included in the one-stage meta-analysis was assessed by comparison between included and unavailable studies and by undertaking the two-stage meta-analysis which including aggregated data from all eligible studies. Within study bias was assessed by the Cochrane Risk of Bias 2 tool^23^ for randomised controlled trials and the Joanna Briggs Institute Case Series tool for single arm studies.^24^

Statistical analyses were conducted in Stata, V.17 (StataCorp) according to an a priori statistical analysis plan.^25^ The review protocol was registered in PROSPERO: CRD42022313730.^26^

## Results

**Figure 1:**
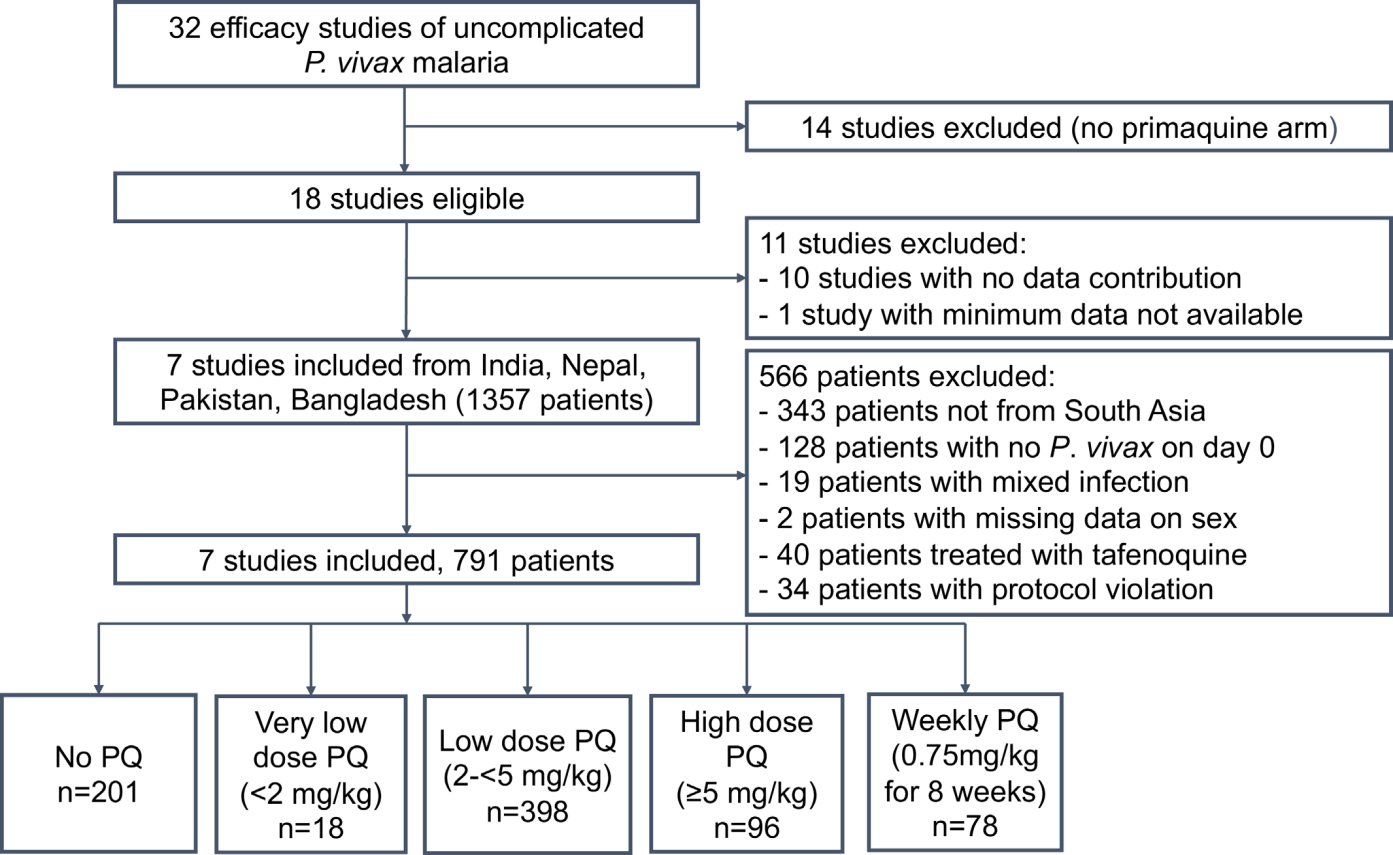
Study flow chart. PQ, primaquine.

The systematic review identified 32 prospective efficacy studies of uncomplicated *P. vivax* malaria from South Asia, of which 14 were excluded as they had no primaquine arm. Of the 18 eligible studies, individual patient data were unavailable for 10 studies^8 9 27–34^ and minimum required data were unavailable for one study^11^ resulting in 7 published studies (38.9%) being included in the individual patient data one-stage meta-analysis (online supplemental tables S1–S5 and figure S1).^35–42^ A total of 566 out of 1357 patients from these studies were excluded, mainly due to enrolment outside of South Asia, treatment with tafenoquine or missing data on parasite density, leaving 791 patients who were eligible for inclusion in the one-stage individual patient data meta-analysis (figure 1).

Of the 791 included patients, the median age was 24.0 years (IQR 16.0–37.0, range 1–80 years), with a median weight of 55.0 kg (IQR 42.0–62.0, range 7–116) (table 1). The majority were male (463, 58.5%) and were treated with chloroquine as schizontocidal treatment (782, 98.9%), with a mean total chloroquine dose of 28.7 mg/kg (SD 7.6, range 12.9–93.8). Patients had planned follow up to 28 days in 3 studies,^35–38^ 180 days in 2 studies^39 40^ and 330–365 days in another 2 studies.^41 42^ Eligible studies that were targeted but not included in the pooled analysis were mainly from India and had longer durations of follow-up and younger patients compared with studies that were included (online supplemental table S6).^8 9 11 27–34^ Risk of bias was low or unclear in all included studies (online supplemental tables S7 and S8).

**Table 1:** Demographic and baseline characteristics

	Total N=791	No primaquine N=201	Daily primaquine			Weekly primaquine (6 mg/kg total over 8 weeks) N=78
			Very low dose total primaquine (<2 mg/kg) N=18	Low-dose total primaquine (2 to <5 mg/kg) N=398	High-dose total primaquine (≥5 mg/kg) N=96	
Sex (%)						
Male	463 (58.5)	74 (36.8)	18 (100)	266 (66.8)	63 (65.6)	42 (53.8)
Female	328 (41.5)	127 (63.2)	0 (0)	132 (33.2)	33 (34.4)	36 (46.2)
Age (years)	24.0 (16.0–37.0)	21.0 (10.0–30.0)	28.5 (25.0–42.0)	30.0 (22.0–42.0)	12.0 (7.0–22.5)	11.0 (8.0–18.0)
Weight (kg)	55.0 (42.0–62.0)	50.0 (25.0–56.0)	90.0 (86.0–103.0)	58.0 (51.0–65.0)	29.5 (18.0–45.0)	30.0 (20.0–48.0)
Presence of fever on day 0 (%)*	559 (94.4)	124 (94.7)	18 (100)	372 (95.6)	40 (81.6)	5 (100%)
Maximum parasitaemia on day 0	2610 (649–7046)	6400 (3180–14000)	1352 (864–5330)	1995 (520–5390)	258 (55–718)	270 (256–810)
Haemoglobin on day 0 (g/L)	126 (20)	120 (18)	144 (13)	129 (20)	122 (20)	126 (18)
Schizontocidal administered (%)						
ACT	9 (1.1)	0 (0)	0 (0)	5 (1.2)	4 (4.2)	0 (0)
CQ	782 (98.9)	201 (100)	18 (100)	393 (98.7)	92 (95.8)	78 (100)
Expected duration of PQ (%)						
14 days	512 (87.2)	0 (0)	18 (100)	398 (100)	96 (100)	0 (0)
7 days	0 (0)	0 (0)	0 (0)	0 (0)	0 (0)	0 (0)
8 weeks	78 (12.8)	0 (0)	0 (0)	0 (0)	0 (0)	78 (100)
Country (%)						
Bangladesh	55 (7.0)	0 (0)	0 (0)	33 (8.3)	22 (22.9)	0 (0)
India	332 (42.0)	30 (14.9)	18 (100)	256 (64.3)	23 (24.0)	5 (6.4)
Nepal	206 (26.0)	101 (50.2)	0 (0)	100 (25.1)	5 (5.2)	0 (0)
Pakistan	198 (25.0)	70 (34.8)	0 (0)	9 (2.3)	46 (47.9)	73 (93.6)

Data are presented as mean (SD) or median (IQR) for continuous measures, and n (%) for categorical measures.

*Data only available for 592 patients.

ACT, artemisinin-based combination therapy; CQ, chloroquine; PQ, primaquine.

There were 201 (25.4%) patients who were not treated with primaquine, 18 (2.3%) were administered a very low total dose of primaquine (<2 mg/kg), 398 (50.3%) were administered low total dose primaquine (2 to <5 mg/kg), 96 (12.1%) were administered high total dose primaquine (≥5 mg/kg) and 78 (9.9%) were administered weekly primaquine (~0.75 mg/kg per week over 8 weeks totalling 6 mg/kg; online supplemental figure S2). The expected duration of the primaquine treatment was 14 days in all 512 patients treated with a daily primaquine dose regimen. All patients treated with daily primaquine regimens had ≥30% G6PD activity. Due to the small number of patients (n=18) in the very low-dose total primaquine group, this group was excluded from efficacy analyses.

### Efficacy of primaquine total mg/kg dose

Between day 7 and 180, 29 recurrences were reported from 713 patients treated without primaquine or with daily primaquine regimens; 25 in patients treated without primaquine, 4 following low total dose primaquine and 0 following high total dose primaquine. Recurrences between day 7 and day 42 were observed in 9 patients treated without primaquine, 2 following low total dose primaquine and 0 following high total dose primaquine. There were 2 recurrences in the 78 patients who were treated with weekly primaquine. The relationship between body weight, mg/kg dose and presence of recurrence between day 7 and 180 is presented in online supplemental figure S3.

**Figure 2:**
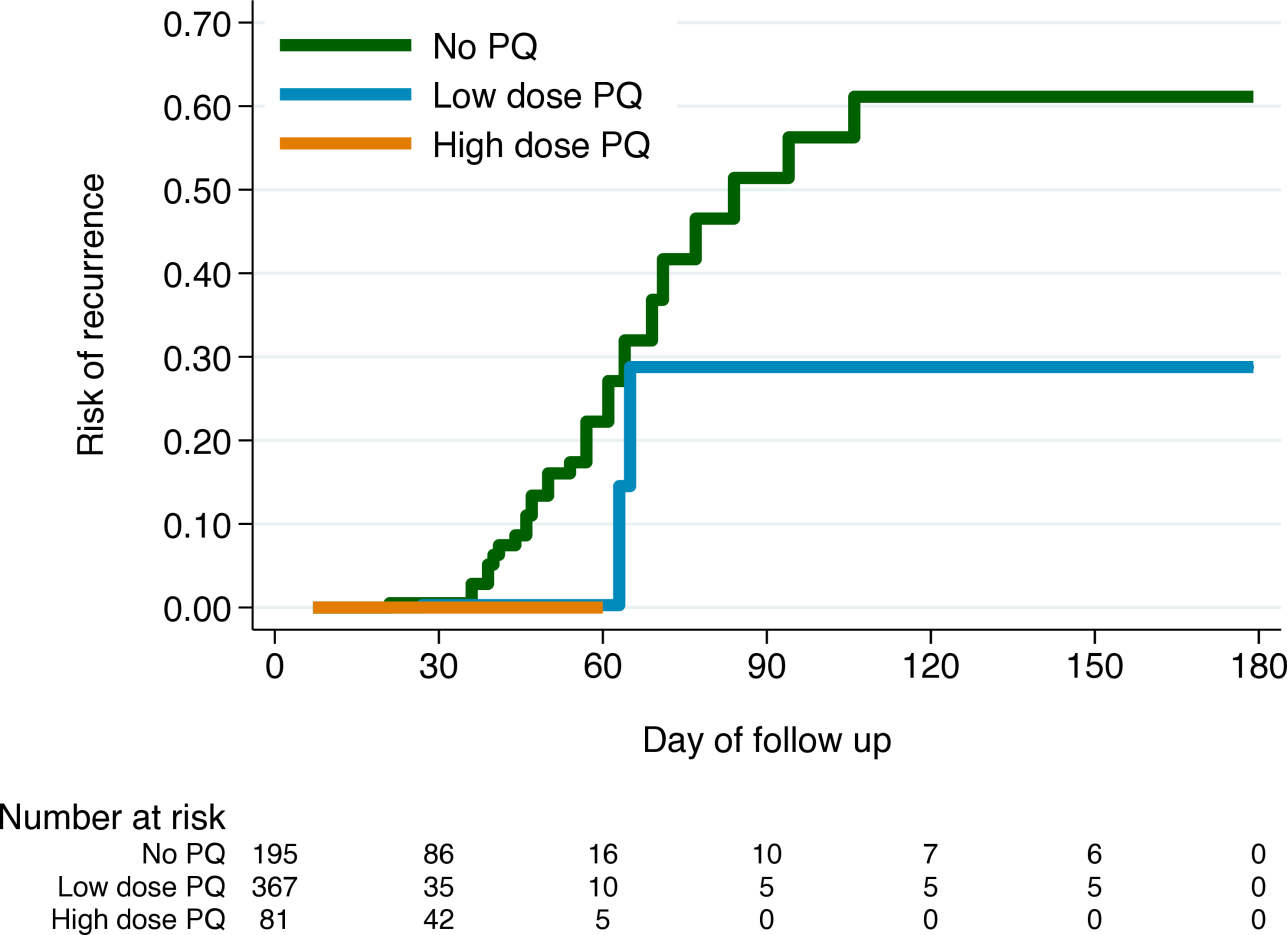
Kaplan-Meier risk of first *Plasmodium vivax* recurrence over 180 days by treatment arm. High dose, ≥5 mg/kg total primaquine dose; low dose, 2 to <5 mg/kg total primaquine dose; PQ, primaquine.

The cumulative risk of recurrence for patients to day 42 was 7.5% (95% CI 3.6% to 15.2%) in patients treated without primaquine compared with 0.2% (95% CI 0.0% to 2.0%) following low total dose primaquine and 0% following high total dose primaquine. The cumulative risk of recurrence for patients at day 180 was 61.1% (95% CI 42.2% to 80.4%) in patients treated without primaquine compared with 28.8% (95% CI 8.2% to 74.1%) in patients with low total dose primaquine and 0% following high total dose primaquine (figure 2); however, despite longer planned follow-up periods no patients receiving high total dose and few patients receiving low total dose primaquine had data available for inclusion past day 60. Results were similar in sensitivity analyses removing one study at a time (online supplemental table S9). The cumulative risk of recurrence at day 42 or day 180 could not be calculated for the 78 patients treated with weekly primaquine, since 76 (97.4%) were only followed until their final day of treatment on day 56.

As the proportional hazards assumption was violated for Cox regression analysis of the risk of recurrence by day 180, due to an unequal proportion of patients being available for analysis beyond day 60 in different treatment groups, the analysis was restricted to days 7–42. Compared with treatment without primaquine, the hazard ratio for first recurrence between days 7 and 42 was 0.4 (95% CI 0.1 to 2.8) following low-dose primaquine. High-dose primaquine was not included in the model as there were no recurrences in this group (table 2).

**Table 2:** Multivariable Cox regression analysis assessing the effect of primaquine treatment arm on risk of *Plasmodium vivax* recurrence between day 7 and day 42

Treatment arm	No of patients	No of recurrences	Adjusted HR* (95% CI)
No primaquine	201	9	Reference
Low-dose total primaquine (2 to <5 mg/kg)	398	2	0.41 (0.06 to 2.77)
High-dose total primaquine (≥5 mg/kg)	96	0	†

*Adjusted for age and sex with random effect for study site.

†Patients with high total dose primaquine were not included in the model as there were no recurrences in this group.

**Figure 3:**
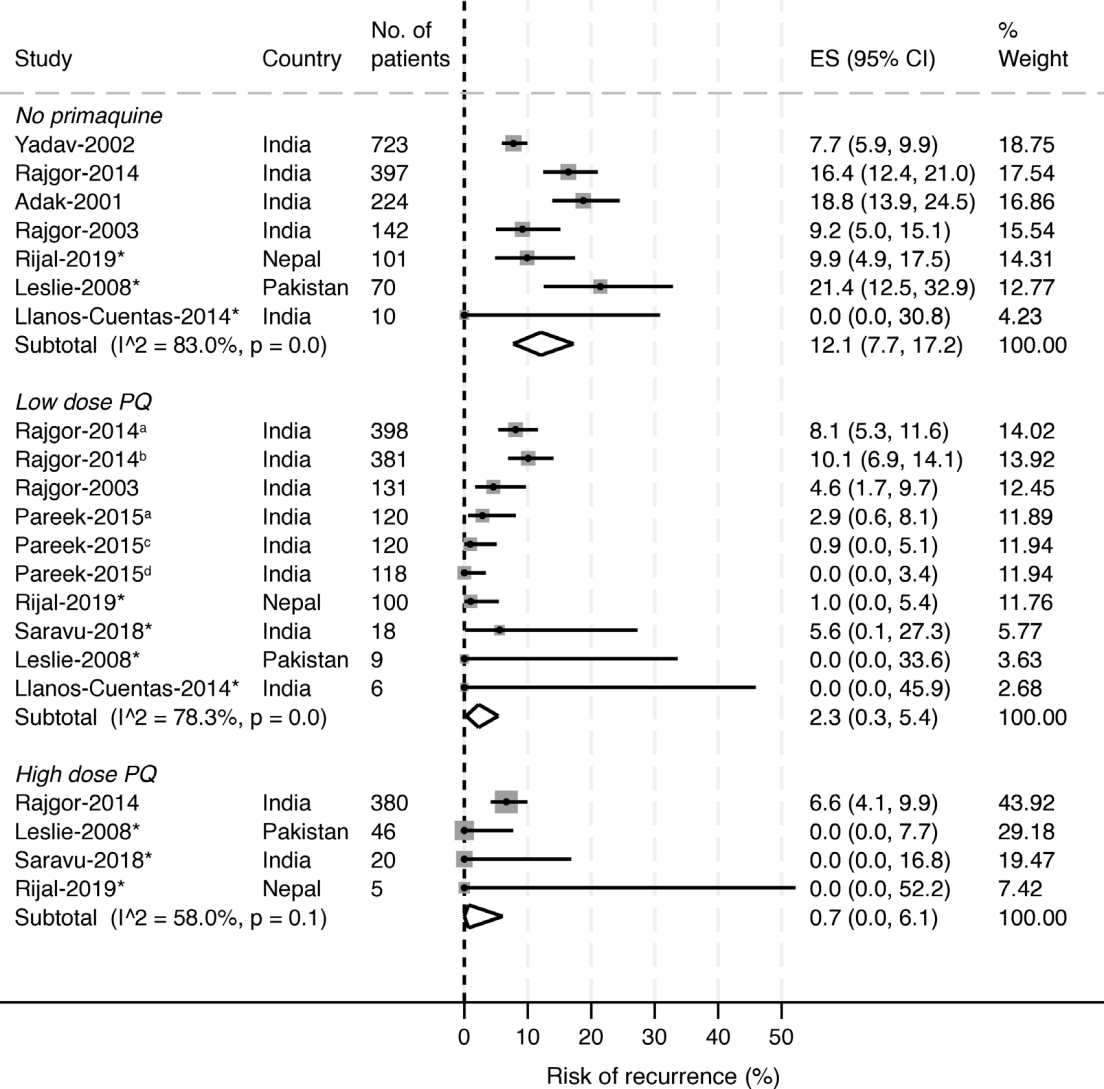
Pooled risk of recurrence by study and treatment arm. Numbers refer to total number of patients available for analysis from the study in that treatment arm. Study-specific treatment estimates were generated from individual patient data where possible (*asterisked studies) and aggregated data from the manuscripts. Pooled estimates for treatment arms were estimated using random-effects meta-analysis with proportions pooled using the Freeman-Tukey double arcsine transformation without adjustment to observed values and with exact methods to calculate confidence intervals. Analysis was restricted to studies with at least 180 days follow-up. a: 15 mg primaquine given for 14 days; b: 30 mg primaquine given for 7 days; c: 30 mg slow release primaquine given for 7 days; d: 15 mg slow release primaquine given for 14 days. ES, effect size; high dose, ≥5 mg/kg total primaquine dose; low dose, 2 to <5 mg/kg total primaquine dose; PQ, primaquine.

From the original systematic review, there were 12 studies with 180 days follow-up or more. After exclusion of three studies (one with a single treatment arm of very low total dose primaquine^31^ and two^11 29^ that did not report the risk of recurrence at day 180), 3529 patients from nine studies were included in a two-stage meta-analysis. This included four studies used in the one-stage meta-analysis^39–42^ and five studies that were targeted for inclusion in the one-stage meta-analysis but for which data were not available^8 9 27 32 33^ (online supplemental figure S4). Risk of bias was low or unclear in all included studies (online supplemental tables S7 and S8). The pooled risk of first *P. vivax* recurrences in 1667 patients treated without primaquine was 12.1% (95% CI 7.7% to 17.2%) compared with 2.3% (95% CI 0.3% to 5.4%) in 1401 patients treated with low total dose primaquine and 0.7% (95% CI 0% to 6.1%) in 451 patients treated with high total dose primaquine (figure 3).

### Safety analysis

The studies did not report any deaths or incidents of blood transfusion. Haematological safety analyses, as part of the one-stage individual patient data meta-analysis, were restricted to the 713 patients treated without primaquine or with daily primaquine; all patients treated with daily primaquine regimens had ≥30% G6PD activity. A day 0 Hb concentration was available for 712 patients with a mean Hb at baseline of 126 g/L (SD 20), including 4 (0.6%) patients with an Hb less than 70 g/L. There were 401 patients with available Hb concentrations between days 1 and 14, of whom 55 (13.7%) had an Hb measured four or more times, 342 (85.3%) had two or three measurements and 4 (1.0%) had a single measurement.

No patients had a fall in Hb of >25% from baseline to <70 g/L, a fall of >50 g/L from day 0 or developed severe anaemia (<50 g/L) (table 3). The absolute and percentage change in Hb from day 0 for different categories of daily doses of primaquine are summarised in table 3. The unadjusted mean absolute change in Hb from day 0 to the minimum Hb between day 1 and 14 was −3 g/L (SD 10) in patients treated without primaquine, −6 g/L (SD 10) following low daily dose primaquine, and −10 g/L (SD 12) following intermediate daily dose primaquine. Data were only available from two patients who received high daily dose primaquine, their fall in Hb was 9 and 6 g/L. Of 399 patients starting with an Hb≥80 g/L at day 0, 2 (0.5%) developed moderate anaemia (<80 g/L to ≥50 g/L) between days 1 and 14, both of whom were treated with intermediate daily dose primaquine.

**Table 3:** Haematological safety outcomes between days 1 and 14 by primaquine daily dose

	No primaquine N=181	Low-dose daily primaquine: <0.375 mg/kg N=150	Intermediate-dose daily primaquine: ≥0.375 to <0.75 mg/kg N=68	High-dose daily primaquine: ≥0.75 mg/kg N=2
Drop in Hb of >25% and to Hb<70 g/L, n/N (%)	0/181 (0)	0/150 (0)	0/68 (0)	0/2 (0)
Drop in Hb of >50 g/L from baseline between days 1 and 14, n/N (%)	0/181 (0)	0/150 (0)	0/68 (0)	0/2 (0)
Absolute change to nadir*, mean (SD) (range) g/L	−3 (10) (−38 to 20)	−6 (10) (−44 to 9)	−10 (12) (−32 to 36)	(−9 to 6)†
Percentage change to nadir*, mean (SD) (range) %	−2.4 (7.8) (−31.4 to 17.8)	−4.3 (6.8) (−26.0 to 7.8)	−7.9 (10.7) (−28.4 to 37.1)	(−9.2 to 5.2)†

*Nadir considered to be lowest measurement between day 1 and day 14.

†As there are only two patients in the high daily dose primaquine group, only the range is given.

Hb, haemoglobin.

No data were available on the presence of vomiting, anorexia, diarrhoea, nausea or abdominal pain following primaquine or acute vomiting within 1 hour of primaquine administration, therefore, no tolerability analysis could be performed.

## Discussion

Primaquine remains the only widely available antimalarial to prevent relapses from liver hypnozoites caused by *P. vivax*, the most common species of malaria in South Asia. However, the relative benefits and drawbacks of the primaquine dose and duration of the currently used regimen of 3.5 mg/kg total dose over 14 days compared with alternative regimens in this region are unclear. This meta-analysis, including individual patient data from 791 patients from seven studies combined with an aggregated two-stage analysis of 3259 patients from nine studies, provides a detailed assessment of efficacy in South Asia, demonstrating good efficacy of low total dose (~3.5 mg/kg) and high total dose (~7 mg/kg) primaquine regimens, without any severe haemolytic events across all regimens. The analysis also highlights the lack of available comparative individual patient data on regimens of different durations and doses with prolonged follow-up and the lack of detailed safety and tolerability data.

Antirelapse efficacy is determined by the total primaquine mg/kg dose administered. Five studies have compared high-dose versus low-dose primaquine, including two from India.^8 40 43–45^ The results of the current meta-analysis support the findings from these studies, suggesting a minimal benefit in raising the total dose of primaquine from 3.5 to 7 mg/kg in preventing relapses in South Asia. This contrasts with other regions of the world, with a recent individual patient data meta-analysis of 6879 patients from all vivax-endemic countries suggesting a benefit of 7 mg/kg total dose primaquine regimens.^10^ WHO currently recommends low total dose primaquine regimens for South Asia and these recommendations are supported by the current meta-analysis.^7 46 47^

Primaquine radical cure regimens are generally administered over 14 days, however, the WHO recently revised treatment guidelines to include a 7-day 3.5 mg/kg total dose primaquine regimen.^7^ Shorter course regimens are expected to improve patient adherence and thus improve antirelapse effectiveness. Two studies from India compared such regimens, although one gave a sustained release formulation of primaquine and the other enrolled patients between 2001 and 2004.^8 9^ All studies in the current individual patient data meta-analysis administered primaquine over 14 days.

In contrast with the studies included in this meta-analysis, most patients treated with primaquine in South Asia are currently not tested for G6PD deficiency. The risk of severe haemolytic events in G6PD deficient individuals is associated with the daily dose. Thus, the increase in the daily primaquine dose with a 7-day regimen from 0.25 mg/kg/day to 0.5 mg/kg/day potentially increases the risk of severe events if G6PD screening is not undertaken. There is a need to further understand the feasibility and risks of a 7-day regimen following G6PD testing compared with a 14-day regimen to better inform national policies in this region.

The current study did not identify any severe haemolytic events in 401 patients treated with or without primaquine. However, the unadjusted absolute reduction in Hb in patients treated with primaquine was greater than those without (6 g/L following low daily dose primaquine, 10 g/L following intermediate daily dose and 3 g/L without primaquine; note only 2 patients received a high daily dose of primaquine). This relationship could be impacted by the baseline Hb concentration. A pooled analysis including 3421 patients from 29 studies found that the nadir Hb was similar in patients with G6PD activity ≥30% treated with and without primaquine at a population level.^48^ A subsequent individual patient data meta-analysis of 5462 patients found a similar risk of haemolysis in patients with ≥30% G6PD activity who were treated with 0.25 mg/kg/day or 0.5 mg/kg/day primaquine compared with patients treated without primaquine.^49^

The relapse behaviour of *P. vivax* varies between regions. *P. vivax* in South Asia has a biphasic phenotype where the duration between initial infection and subsequent relapse is generally either around 1–2 months or >6 months.^50–54^ The current one-stage meta-analysis was limited by relatively few patients treated with low-dose total primaquine and no patients treated with high-dose total primaquine having data available beyond 60 days. Furthermore, since few of the included studies had prolonged follow-up beyond 6 months the relative antirelapse efficacy of low total dose and high total dose primaquine regimens could not be compared against late (>6 months) vivax malaria relapses. Even if studies with prolonged follow-up had been available, assessment of antirelapse efficacy would be potentially confounded by an increasing proportion of recurrences being due to reinfection. The two-stage meta-analysis demonstrated good antirelapse efficacy of low total dose and high total dose primaquine regimens within 180 days.

Gastrointestinal symptoms are a recognised side effect of primaquine, although they are also associated with acute malaria and schizontocidal drugs. Their association with primaquine dose has not been clearly defined, although it appears that the risk of developing them may be reduced by taking primaquine with food.^55 56^ No data were available to assess the risk of gastrointestinal symptoms with different primaquine regimens in the current analysis. Future studies in the region should consider assessing gastrointestinal symptoms and recording whether primaquine was coadministered with food or not.

This study was limited by inclusion of only 39% of eligible studies in the individual patient data meta-analysis, although 7 of 11 studies that were not available to share individual patient data were published before 2010 and 10 of 11 studies that were not included were from India. The routine collation and sharing of data from future clinical efficacy studies would enable greater power to investigate differences in the comparative risks and benefits of antirelapse regimens going forward. Primaquine mg/kg dosing was derived from study protocols in 91% of patients. These mg/kg doses may have differed to the actual doses administered. In addition, there was a significant difference in the population that received high-dose total primaquine compared with low-dose primaquine or were treated without primaquine. Patients receiving high-dose primaquine were younger, however, their young age would have been expected to put them at greater risk of recurrence^10^ and this is, therefore, unlikely to have impacted the results of the analysis. Although our analysis aimed to evaluate the antirelapse efficacy of primaquine, it is not possible to determine whether recurrent parasitaemia arises from recrudescences (schizontocidal treatment failures), relapses (hypnozoiticidal treatment failures) or reinfections. For this reason, the risk of recurrence rather than relapse was used as the primary efficacy endpoint; which is confounded by reinfection and background malaria endemicity.

## Conclusions

In summary, the risk of *P. vivax* recurrence was low at day 180 following low and high total dose primaquine, supporting the current recommendation for low total dose primaquine regimens in South Asia. In general, there was a lack of available individual patient data to assess efficacy, tolerability and safety across this region, with no individual patient data available to assess the relative efficacy of 7-day vs 14-day primaquine regimens or the effect of primaquine daily dose on gastrointestinal symptoms. No severe haemolytic events were identified following any treatment.

As countries in the region progress towards malaria elimination by 2030, relapsing malaria caused by hypnozoites poses a major challenge to achieving this goal. The introduction of shorter course regimens may improve adherence and effectiveness and fast track this progress. However, implementation of such courses needs to occur in conjunction with routine G6PD testing at the point of care given the potential for an increased haemolytic risk in G6PD deficient patients with higher daily dose primaquine regimens.

## Data Availability

Data are available on reasonable request. The data that support the findings of this study are available for access via the WorldWide Antimalarial Resistance Network (WWARN.org). Requests for access will be reviewed by a Data Access Committee to ensure that the use of data protects the interests of the participants and researchers according to the terms of ethics approval and principles of equitable data sharing. Requests can be submitted by email to malariaDAC@iddo.org via the Data Access Form available at WWARN.org/accessing-data. The WWARN is registered with the Registry of Research Data Repositories (re3data.org).
