# Supplementary material for: Safety and efficacy of primaquine in patients with Plasmodium vivax malaria from South Asia: a systematic review and individual patient data meta-analysis
Source: BMJ Glob Health. 2023 Dec 20;8(12):e012675. doi: 10.1136/bmjgh-2023-012675 (PMC10749047; doi:10.1136/bmjgh-2023-012675)
Supplement: Supplementary data [file bmjgh-2023-012675supp001.pdf]

ADDITIONAL FILE 1

Verma R *et al*, Safety and efficacy of primaquine in patients with *P. vivax* malaria from South Asia: A systematic review and individual patient data meta-analysis

	Page
**Checklist S1.** PRISMA-IPD	2
**Box S1.** Search Strategy	7
**Table S1.** Studies included in the one stage individual patient data meta-analysis	8
**Table S2.** Study testing and inclusion criteria	9
**Table S3.** Study sites included in the one stage individual patient data meta-analysis	10
**Table S4.** Reasons for studies not being included in the one stage efficacy meta-analysis	11
**Table S5.** Studies targeted for the one stage efficacy analysis but not included	12
**Table S6:** Comparison of baseline characteristics between studies included and studies targeted for the one stage meta-analysis	13
**Table S7:** Risk of bias assessment in randomised controlled studies in one or two stage meta-analyses	14
**Table S8:** Risk of bias assessment in single arm observational studies in one or two stage meta-analyses	15
**Table S9:** Sensitivity analysis for cumulative risk of first *P. vivax* recurrence between day 7 to 42 and between day 7 to 180	16
**Figure S1.** Location of study sites in efficacy analysis	17
**Figure S2.** Mg/kg total dose of primaquine administered	18
**Figure S3.** Mg/kg drug dosing of primaquine by body weight	19
**Figure S4.** Flowchart for two stage meta-analysis	20

Verma R, *et al. BMJ Glob Health* 2023; 8:e012675. doi: 10.1136/bmjgh-2023-012675

Checklist S1. PRISMA-IPD Checklist of items to include when reporting a systematic review and meta-analysis of individual participant data (IPD)

PRISMA-IPD Section/topic	Item No	Checklist item	Reported on page
**Title**			
Title	1	Identify the report as a systematic review and meta-analysis of individual participant data.	1
**Abstract**			
Structured summary	2	Provide a structured summary including as applicable:	
		**Background**: state research question and main objectives, with information on participants, interventions, comparators and outcomes.	4
		**Methods**: report eligibility criteria; data sources including dates of last bibliographic search or elicitation, noting that IPD were sought; methods of assessing risk of bias.	4
		**Results**: provide number and type of studies and participants identified and number (%) obtained; summary effect estimates for main outcomes (benefits and harms) with confidence intervals and measures of statistical heterogeneity. Describe the direction and size of summary effects in terms meaningful to those who would put findings into practice.	4
		**Discussion:** state main strengths and limitations of the evidence, general interpretation of the results and any important implications.	4,5
		**Other:** report primary funding source, registration number and registry name for the systematic review and IPD meta-analysis.	5
**Introduction**			
Rationale	3	Describe the rationale for the review in the context of what is already known.	8
Objectives	4	Provide an explicit statement of the questions being addressed with reference, as applicable, to participants, interventions, comparisons, outcomes and study design (PICOS). Include any hypotheses that relate to particular types of participant-level subgroups.	8

Methods			
Protocol and registration	5	Indicate if a protocol exists and where it can be accessed. If available, provide registration information including registration number and registry name. Provide publication details, if applicable.	11, Ref 28
Eligibility criteria	6	Specify inclusion and exclusion criteria including those relating to participants, interventions, comparisons, outcomes, study design and characteristics (e.g. years when conducted, required minimum follow-up). Note whether these were applied at the study or individual level i.e. whether eligible participants were included (and ineligible participants excluded) from a study that included a wider population than specified by the review inclusion criteria. The rationale for criteria should be stated.	9-10, ref 27,28
Identifying studies - information sources	7	Describe all methods of identifying published and unpublished studies including, as applicable: which bibliographic databases were searched with dates of coverage; details of any hand searching including of conference proceedings; use of study registers and agency or company databases; contact with the original research team and experts in the field; open adverts and surveys. Give the date of last search or elicitation.	9, Box S1
Identifying studies - search	8	Present the full electronic search strategy for at least one database, including any limits used, such that it could be repeated.	9 and Box S1
Study selection processes	9	State the process for determining which studies were eligible for inclusion.	9,10 and Box S1
Data collection processes	10	Describe how IPD were requested, collected and managed, including any processes for querying and confirming data with investigators. If IPD were not sought from any eligible study, the reason for this should be stated (for each such study).	Table S1, S2 and S5
		If applicable, describe how any studies for which IPD were not available were dealt with. This should include whether, how and what aggregate data were sought or extracted from study reports and publications (such as extracting data independently in duplicate) and any processes for obtaining and confirming these data with investigators.	
Data items	11	Describe how the information and variables to be collected were chosen. List and define all study level and participant level data that were sought, including baseline and follow-up information. If applicable, describe methods of standardising or translating variables within the IPD datasets to ensure common scales or measurements across studies.	10 and Box S1
IPD integrity	A1	Describe what aspects of IPD were subject to data checking (such as sequence generation, data consistency and completeness, baseline imbalance) and how this was done.	Ref 19

Risk of bias assessment in individual studies.	12	Describe methods used to assess risk of bias in the individual studies and whether this was applied separately for each outcome. If applicable, describe how findings of IPD checking were used to inform the assessment. Report if and how risk of bias assessment was used in any data synthesis.	11-12 and Ref 24 and 27
Specification of outcomes and effect measures	13	State all treatment comparisons of interests. State all outcomes addressed and define them in detail. State whether they were pre-specified for the review and, if applicable, whether they were primary/main or secondary/additional outcomes. Give the principal measures of effect (such as risk ratio, hazard ratio, difference in means) used for each outcome.	11
Synthesis methods	14	Describe the meta-analysis methods used to synthesise IPD. Specify any statistical methods and models used. Issues should include (but are not restricted to):<br>• Use of a one-stage or two-stage approach.<br>• How effect estimates were generated separately within each study and combined across studies (where applicable).<br>• Specification of one-stage models (where applicable) including how clustering of patients within studies was accounted for.<br>• Use of fixed or random effects models and any other model assumptions, such as proportional hazards.<br>• How (summary) survival curves were generated (where applicable).<br>• Methods for quantifying statistical heterogeneity (such as I^2 and t^2).<br>• How studies providing IPD and not providing IPD were analysed together (where applicable).<br>• How missing data within the IPD were dealt with (where applicable).	11-12
Exploration of variation in effects	A2	If applicable, describe any methods used to explore variation in effects by study or participant level characteristics (such as estimation of interactions between effect and covariates). State all participant-level characteristics that were analysed as potential effect modifiers, and whether these were pre-specified.	12
Risk of bias across studies	15	Specify any assessment of risk of bias relating to the accumulated body of evidence, including any pertaining to not obtaining IPD for particular studies, outcomes or other variables.	Box S1, Ref. 24,27
Additional analyses	16	Describe methods of any additional analyses, including sensitivity analyses. State which of these were pre-specified.	12 and Table S9
Results			

Study selection and IPD obtained	17	Give numbers of studies screened, assessed for eligibility, and included in the systematic review with reasons for exclusions at each stage. Indicate the number of studies and participants for which IPD were sought and for which IPD were obtained. For those studies where IPD were not available, give the numbers of studies and participants for which aggregate data were available. Report reasons for non-availability of IPD. Include a flow diagram.	13-15 , Tables S1, S4 and S5
Study characteristics	18	For each study, present information on key study and participant characteristics (such as description of interventions, numbers of participants, demographic data, unavailability of outcomes, funding source, and if applicable duration of follow-up). Provide (main) citations for each study. Where applicable, also report similar study characteristics for any studies not providing IPD.	13, Table S1, S2 and S5
IPD integrity	A3	Report any important issues identified in checking IPD or state that there were none.	
Risk of bias within studies	19	Present data on risk of bias assessments. If applicable, describe whether data checking led to the up-weighting or down-weighting of these assessments. Consider how any potential bias impacts on the robustness of meta-analysis conclusions.	Tables S7 and S8
Results of individual studies	20	For each comparison and for each main outcome (benefit or harm), for each individual study report the number of eligible participants for which data were obtained and show simple summary data for each intervention group (including, where applicable, the number of events), effect estimates and confidence intervals. These may be tabulated or included on a forest plot.	18 and Figure 3
Results of syntheses	21	Present summary effects for each meta-analysis undertaken, including confidence intervals and measures of statistical heterogeneity. State whether the analysis was pre-specified, and report the numbers of studies and participants and, where applicable, the number of events on which it is based.	14-20
		When exploring variation in effects due to patient or study characteristics, present summary interaction estimates for each characteristic examined, including confidence intervals and measures of statistical heterogeneity. State whether the analysis was pre-specified. State whether any interaction is consistent across trials.	
		Provide a description of the direction and size of effect in terms meaningful to those who would put findings into practice.	
Risk of bias across studies	22	Present results of any assessment of risk of bias relating to the accumulated body of evidence, including any pertaining to the availability and representativeness of available studies, outcomes or other variables.	Ref. 24,27 Table S1, S2, S5 and S6

Additional analyses	23	Give results of any additional analyses (e.g. sensitivity analyses). If applicable, this should also include any analyses that incorporate aggregate data for studies that do not have IPD. If applicable, summarise the main meta-analysis results following the inclusion or exclusion of studies for which IPD were not available.	Tables S6 and S9
**Discussion**			
Summary of evidence	24	Summarise the main findings, including the strength of evidence for each main outcome.	22-25
Strengths and limitations	25	Discuss any important strengths and limitations of the evidence including the benefits of access to IPD and any limitations arising from IPD that were not available.	22-25
Conclusions	26	Provide a general interpretation of the findings in the context of other evidence.	6, 24-25
Implications	A4	Consider relevance to key groups (such as policy makers, service providers and service users). Consider implications for future research.	24-25
**Funding**			
Funding	27	Describe sources of funding and other support (such as supply of IPD), and the role in the systematic review of those providing such support.	25

© Reproduced with permission of the PRISMA IPD Group, which encourages sharing and reuse for non-commercial purposes

Box S1. Search strategy

Search strategy

All prospective antimalarial efficacy studies of uncomplicated *P. vivax* with a minimum of 28 days follow up, published between Jan 1, 2000 and August 23, 2021 were identified by the application of the key terms (listed below) through Medline (Pubmed), Web of Science, Embase and the Cochrane Central. Abstracts of all references containing any mention of antimalarial drugs were manually checked to confirm prospective clinical trials, with review of full text when needed. Studies on prevention, prophylaxis, reviews, animal studies, patients with severe malaria, where schizontocidal treatment was unsupervised or where data were extracted retrospectively from medical records outside of a planned trial were excluded. Inclusion criteria included studies undertaken in South Asia including Bangladesh, Bhutan, India, Nepal, Pakistan, Sri Lanka. Studies were included if they had at least one treatment arm with primaquine commencing within 7 days of schizontocidal treatment, had data on age, sex and parasitemia on day 0, data on schizontocidal and primaquine dosing and reported parasite presence or absence during follow up. The year of the study was taken as the year in which the paper was published, although the start and end date of patient enrolment were also recorded. The review process was undertaken by two independent investigators who also performed data extraction (RV and RJC), and the original review process is documented in more detail in, Commons RJ, Thriemer K, Humphreys G, et al. *Int J Parasitol Drugs Drug Resist*. 2017;7(2):181-190 [17].

Key terms:

Literature search (conducted March 2022) with the following key terms (version undertaken in Pubmed): (malaria OR plasmod*) AND (amodiaquine OR atovaquone OR artemisinin OR arteether OR artesunate OR artemether OR artemotil OR azithromycin OR artekin OR chloroquine OR chlorproguanil OR cycloguanil OR clindamycin OR coartem OR dapsone OR dihydroartemisinin OR duo-cotecxin OR doxycycline OR halofantrine OR lumefantrine OR lariam OR malarone OR mefloquine OR naphthoquine OR naphthoquinone OR piperaquine OR primaquine OR proguanil OR pyrimethamine OR pyronaridine OR quinidine OR quinine OR riamet OR sulphadoxine OR tetracycline OR tafenoquine).

Table S1. Studies included in the one stage individual patient data meta-analysis

Author-year	Country	Recruitment Period	Age range (years)	Follow up (days)	Included treatment arms*	PQ supervision	Randomised	Patients available	Included in GI tolerability analysis	Included in haematology analysis	Included in efficacy analysis
Leslie-2008 (42)	Pakistan	2004 – 2006	4-80	330	Cq, Cq_Pq_7.0_14d_D0	Full	Yes	198	No	Yes	Yes
Llanos Cuentas-2013 (39)	India	2011-2013	>16	180	Cq, Cq_Pq_3.4_14d, Cq_Tq	Partial	Yes	16	No	Yes	Yes
Rishikesh-2015 (36,37)	India	2012-2014	18-76	28#	Cq_Pq_3.5_14d	Partial	No	117	No	No	Yes
Ley-2016 (38)	Bangladesh	2014 – 2015	1-66	30	Cq_Pq_3.5_14d_D2	No	No	55	No	Yes	Yes
Saravu-2016 (35, 36)	India	2012-2015	> 18	28#	Cq_Pq_3.5_14d, D0	No	No	161	No	No	Yes
Saravu-2018 (40)	India	2012-2014	>18	180	Cq/ACT_Pq_7_14d, Cq/ACT_Pq_3.5_14d, D2	Partial	Yes	38	No	Yes	Yes
Rijal-2019 (41)	Nepal	2015 - 2016	5-75	365	Cq, Cq_Pq_3.5_14d_D0	Partial	Yes	206	Yes	Yes	Yes

ACT – artemisinin-based combination treatment; Cq – chloroquine; GI – gastrointestinal; PQ/Pq – primaquine; Primaquine treatment was classified as *supervised* if all doses were directly observed, *partially supervised* if >1 dose but not all doses were observed, and *not-supervised* if ≤1 dose was observed; *Treatment code describes (schizontocidal drug)_(hypnozoitocidal drug)_(total primaquine dose)_(duration of primaquine treatment eg 14d = 14 days)_(primaquine start day); # Reference 36 is a follow up study up to 450 days including some of the patient cohort in references 35 and 37.

Verma R, *et al. BMJ Glob Health* 2023; 8:e012675. doi: 10.1136/bmjgh-2023-012675

Table S2. Study testing and inclusion criteria

Author-year	Hb exclusion criteria	G6PD testing undertaken	G6PD activity included	G6PD data available	Days symptom checklist undertaken and data available	Days Hb/Hct measured and data available
Leslie-2008 (42)	Hb <7 g/dL	Colorimetric test	All activities (if deficient treated with 8 weekly 0.75 mg/kg PQ)	Yes	-	0, 7, 14
Llanos-Cuentas-2013 (39)	Hb< 7 g/dL	Spectrophotometric semiquantitative assay (Trinity Biotech. Bray, County Wicklow, Ireland or Pointe Scientific, Cnaton, MI, USA	All activities (excluded if <70% G6PD activity)	Yes	0	0, 3, 5, 8, 11, 15, 22, 29
Rishikesh-2015 (36,37)*	No exclusion stated	Spectrophotometry	All activities included (if deficient treated with 8 weekly 0.75 mg/kg PQ)	No	0	0
Ley-2016 (38)	Hb <8 g/dL	Spectrophotometry	All activities (if deficient not given PQ)	Yes	-	0, 2, 9
Saravu-2016 (35,36)*	No exclusion stated	Spectrophotometry	All activities (if deficient treated with 8 weekly 0.75 mg/kg PQ)	Yes	0	0, 7, 14, 28
Saravu-2018 (40)	No exclusion stated	Spectrophotometry	All activities (if deficient not given PQ)	No	0	0, 7, 14, 28
Rijal-2019 (41)	No exclusion stated	Carestart RDT	≥30% activity	Yes	0	0, 1, 3, 7

Hb – haemoglobin; Hct – haematocrit. * Reference 36 is a follow up study up to 450 days including some of the patient cohort in references 35 and 37.

Verma R, *et al. BMJ Glob Health* 2023; 8:e012675. doi: 10.1136/bmjgh-2023-012675

Table S3. Study sites included in the one stage individual patient data meta-analysis

Author-year	Study site	Country	Latitude	Longitude	Year Start	Year End	MAP Incidence rate (per 1000 persons)	Transmission intensity*
Leslie-2008 (42)	Adizai	Pakistan	33.79	71.58	2004	2006	2.48	Moderate
Leslie-2008 (42)	Baghicha	Pakistan	34.23	72.16	2004	2006	2.48	Moderate
Leslie-2008 (42)	Khagan	Pakistan	34.54	73.32	2004	2006	2.48	Moderate
Llanos-Cuentas-2014 (39)	Lucknow	India	26.85	80.94	2011	2013	0.16	Low
Llanos-Cuentas-2014 (39)	Bikaner	India	28.02	73.31	2011	2013	0.56	Low
Llanos-Cuentas-2014 (39)	Chennai	India	13.08	80.27	2011	2013	1.18	Low
Rishikesh-2015 (36,37)	Manipal	India	12.96	77.65	2012	2014	2.5	Moderate
Ley-2016 (38)	Alikadam	Bangladesh	21.65	92.31	2014	2015	0.59	Low
Saravu-2016 (35, 36)	Udupi	India	13.34	74.74	2012	2015	2.5	Moderate
Saravu-2018 (40)	Udupi	India	13.34	74.74	2017	2018	2.5	Moderate
Rijal-2019 (41)	Jhapa	Nepal	26.55	87.89	2016	2016	0.12	Low
Rijal-2019 (41)	Kailali	Nepal	28.83	80.90	2016	2016	0.22	Low

MAP – malaria Atlas Project; *Transmission intensity is classified as low (an incidence rate of <1 per 1000 persons), moderate (1 to <10 per 1000 persons), high (≥10 per 1000 persons).

Verma R, *et al. BMJ Glob Health* 2023; 8:e012675. doi: 10.1136/bmjgh-2023-012675

Table S4. Reasons for studies not being included in the one stage efficacy meta-analysis

**Reason**	**Efficacy analysis**	
	**Number of studies**	**Studies**
Data not available	5	8, 27, 28, 33-34
Investigators unable to be contacted	1	29
No response from investigators	4	9, 30-32
Minimum data not available	1	11

Table S5. Studies targeted for the one stage efficacy individual patient data meta-analysis but not included

First Author	Treatment Arms	Number of Sites	Country	Follow up (days)	Randomised	Recruitment period	Treatment arms	Pv patients enrolled	Treated with PQ	Female (%)	Mean Age (SD)	Median Age (range)	Reasons for exclusion
Adak-2001 (27)	3	1	India	365	Yes	Not stated	Cq; Cq_Pq_1.25_5d_D3; Cq_Bq	663	220	Not stated	Not stated		Data not available
Dua-2001 (31)	1	4	India	540	No	1987-2000	Cq_Pq_1.25_5d_D2	5541	5541	Not stated	Not stated		No response from investigators
Mohapatra-2002 (29)	1	1	India	365	No	1998-2000	Cq_Pq_3.5_14d_DX	110	110	36.4	Not stated		Unable to be contacted
Yadav-2002 (32)	2	1	India	365	Yes	1988-1991	Cq; Cq_Pq_1.25_5d_D2	1482	759	Not stated	Not stated		No response from investigators
Rajgor-2003 (33)	2	1	India	180	Yes	1998-2000	Cq; Cq_Pq_3.5_14d_D4	273	131	12.1	Not stated		Data not available
Leslie-2004 (11)	3	1	Pakistan	270	Yes	2000-2001	Cq; Cq_Pq_3.5_14d_D0(sup); Cq_Pq_3.5_14d_D0(unsup)	595	383	50.7	12.9 (-)		Minimum data not available
Dunne-2005 (30)	2	6	India	28	Yes	1998-2001	Cq _Pq_3.5_14d_D3 ; AZ_Pq_3.5_14d_D3	200	102	20	32		No response from investigators
Ganguly-2013 (34)	2	1	India	42	Yes	2011-2012	Cq; Cq_Pq_3.5_14d_D0	250	125	10.8	25.2 (-)		Data not available
Rajgor-2014 (8)	4	1	India	180	Yes	Not stated	Cq; Cq_Pq_3.5_14d_D4; Cq_Pq_3.5_7d_D4; Cq_Pq_7.0_14d_D4	1556	1159	4.8	31.2 (-)		Data not available
Pareek-2015 (9)	3	8	India	180	Yes	Not stated	Cq_Pq_3.5_14d_D3; Cq_Pq_3.5_14d_D3; Cq_Pq_3.5_7d_D3	358	358	17.3	Not stated	20	No response from investigators
Valecha-2016 (28)	2	9	India	42	Yes	2011-2013	Cq_Pq_3.5_14d_D3; ArtmPip_Pq_3.5_14d_D3	317	317	8.2	33.7 (13.5)		Data not available

Artm – arterolane maleate; AZ – azithromycin; Bq – bulaquine; Cq – chloroquine; Pip – piperaquine; PQ/Pq – primaquine; Pv – *P. vivax*; SD – standard deviation; Tnd – tinidazole; Tq – tafenoquine; *Treatment code describes (schizontocidal drug)_(hypnozoitocidal drug)_(total primaquine dose)_(duration of primaquine treatment eg 14d = 14 days)_(primaquine start day) (supervision status)

Table S6: Comparison of baseline characteristics between studies included and studies targeted for the one stage meta-analysis

Characteristic	Included studies<br>N = 7	Targeted studies that were not included<br>N = 11
**Year of Enrolment**		
Pre 2010	1 (14.2%)	7 (63.6%)
2010 or later	6 (85.7%)	4 (36.4%)
**Countries**		
India	4 (57.0%)	10 (90.9%)
Bangladesh	1 (14.2%)	0 (0%)
Pakistan	1 (14.2%)	1 (9.1%)
Nepal	1 (14.2%)	0 (0%)
**Age (years), median (inter-quartile range)**	21.3 (16-35.6)	31.2 (20-31.2)*
**Female, %**	34.8%	16.5%#

Age and female percentage of targeted studies frequency weighted according to number of patients treated with chloroquine alone (No primaquine) and different dosages of primaquine. Year of the enrolment defined as the year study enrolment completed. Age, and female percentage of targeted studies calculated using frequency weighted mean or median according to number of patients. * Mean or median age not available for 5 studies. # Percentage not available for 3 studies.

Table S7. Risk of bias assessment in randomised controlled studies in one or two stage meta-analyses

Author-year	Bias from randomisation	Bias due to deviation from intervention	Bias from missing outcome	Bias in measurement of the outcome	Bias in selection of the reported results	Overall bias	Follow up to 180 days
Adak-2001 (27)	Green	Green	Green	Green	Green	Green	Green
Yadav-2002 (32)	Orange	Orange	Green	Green	Orange	Orange	Green
Rajgor-2003 (33)	Green	Green	Orange	Green	Green	Orange	Green
Leslie-2008 (42)	Orange	Green	Green	Green	Orange	Orange	Green
Llanos-Cuentas-2014 (39)	Green	Green	Green	Green	Green	Green	Green
Rajgor-2014 (8)	Green	Green	Orange	Green	Green	Orange	Green
Pareek-2015 (9)	Green	Green	Green	Green	Orange	Orange	Green
Saravu-2018 (40)	Green	Green	Orange	Green	Orange	Orange	Green
Rijal-2019 (41)	Green	Green †	Orange	Green	Orange	Orange	Green

Green – low risk of bias; Red – high risk of bias; Orange – unclear risk of bias; Grey – not applicable; Assessed according to the Cochrane Risk of Bias 2 tool for randomised controlled trials (23); † Study analysed per protocol but all data available for these meta-analyses; PQ – primaquine.

Table S8. Risk of bias assessment in single arm observational studies in one or two stage meta-analyses

Author-year	Clear criteria for inclusion	Condition measured in reliable way	Valid methods for condition	Consecutive inclusion	Complete inclusion	Demographics reported	Clinical information reported	Outcomes reported	Site description	Analysis appropriate	Follow up to 180 days
Rishikesh-2015 (37)											
Ley-2016 (38)											
Saravu-2016 (35)											

Green – yes (low risk of bias); Red – no (higher risk of bias); Orange – unclear; Grey – not applicable; Assessed according to the Joanna Briggs Institute Case Series tool (24) for single arm studies; The appropriateness of analysis was considered appropriate for all studies given that the individual patient data were re-analysed as part of these meta-analyses; PQ – primaquine.

Verma R, *et al. BMJ Glob Health* 2023; 8:e012675. doi: 10.1136/bmjgh-2023-012675

Table S9: Sensitivity analysis for cumulative risk of first *P. vivax* recurrence between day 7 to 42 and between day 7 to 180 for the patients receiving different dosage of primaquine

Variable	Range of cumulative risk (%)	Coefficient of Variation (%)
**Day 42**		
No primaquine	2.28-11.66	26.58
Low dose primaquine	0.00-0.45	35.39
High dose primaquine	0.00-0.00	
**Day 180**		
No primaquine	46.68-79.62	13.57
Low dose primaquine	16.97-50.14	28.67
High dose primaquine	0.00-0.00	

Sensitivity analyses were generated by removing one study site at a time from the Kaplan-Meier cumulative risk calculation. The coefficient of variation is calculated as standard deviation divided by the mean of the estimates of the $\log_e$(Cumulative Risk). Low dose = 2 - <5 mg/kg; High dose = ≥5 mg/kg.

Verma R, *et al. BMJ Glob Health* 2023; 8:e012675. doi: 10.1136/bmjgh-2023-012675

Figure S1: Location of study sites in efficacy analysis

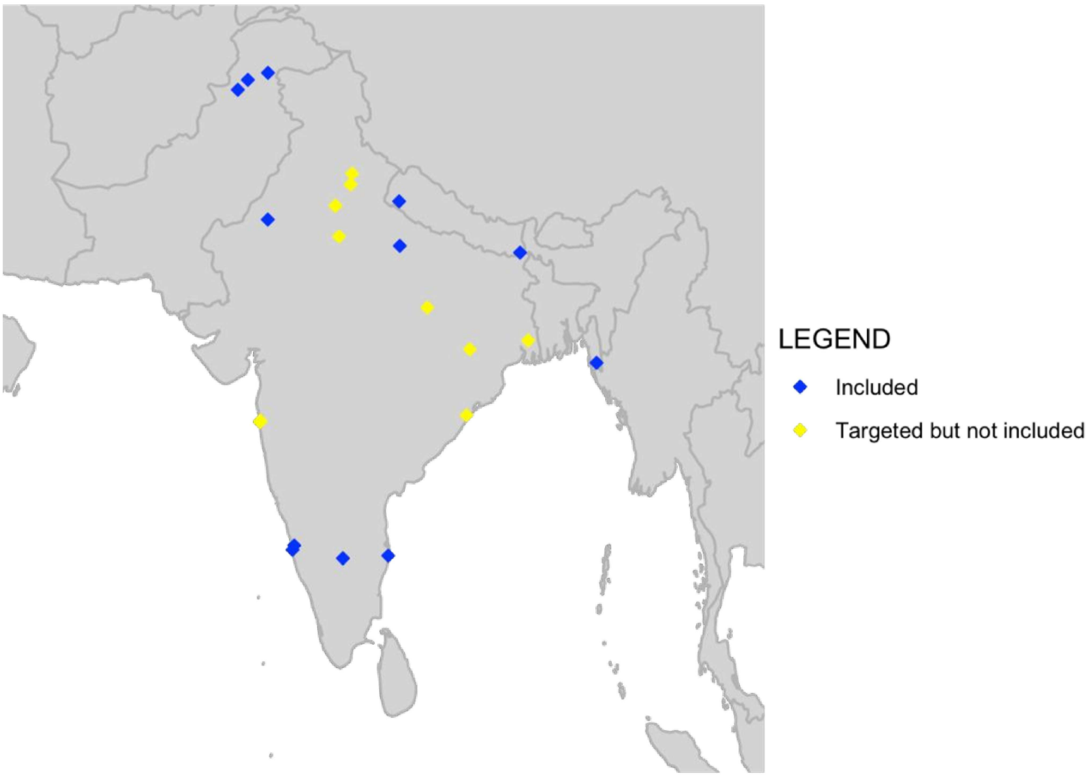

Figure S2: Mg/kg total dose of total primaquine administered (n=512)

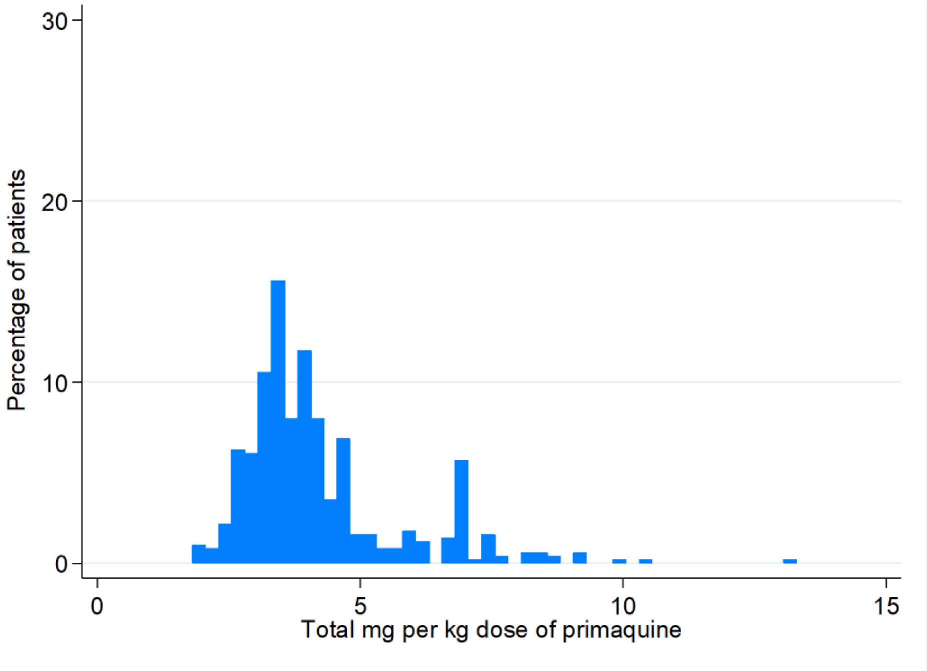

Figure S3: Mg/kg drug dosing of primaquine by body weight (n=512)

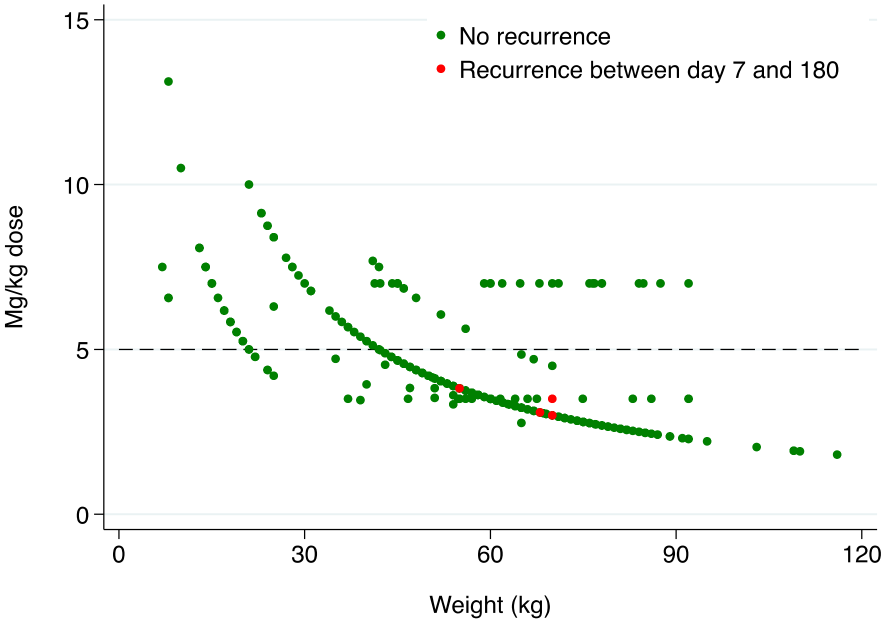

Figure S4: Flowchart for two stage meta-analysis

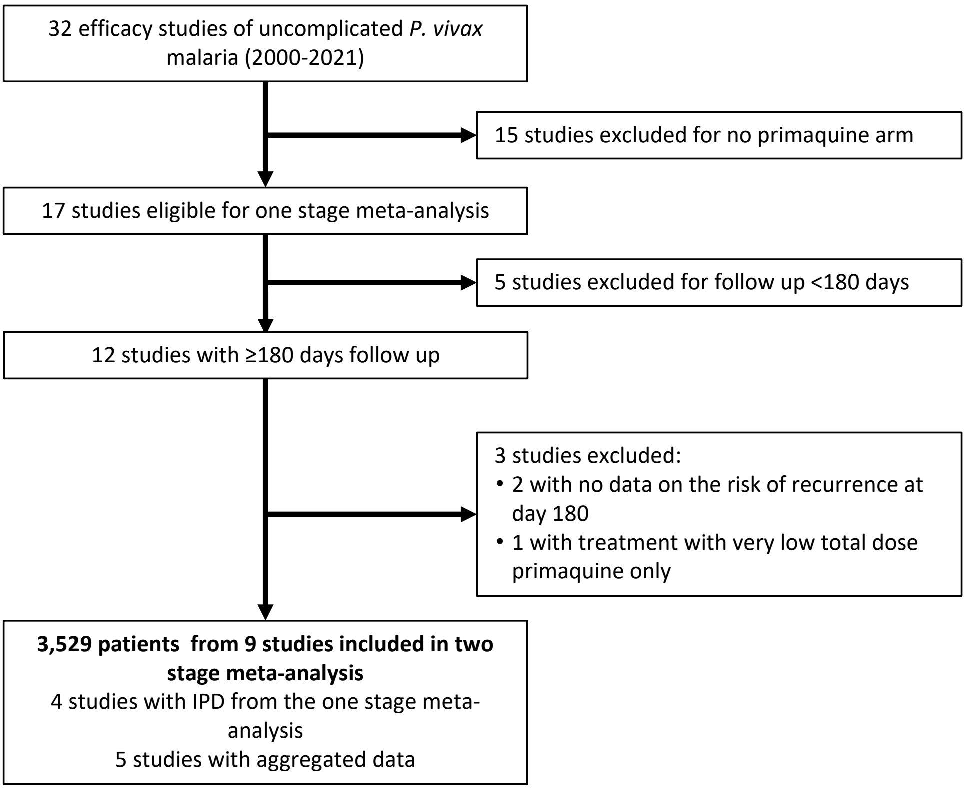

IPD – individual patient data
